# Prediction of malaria transmission drivers in *Anopheles* mosquitoes using artificial intelligence coupled to MALDI-TOF mass spectrometry

**DOI:** 10.1038/s41598-020-68272-z

**Published:** 2020-07-09

**Authors:** Cécile Nabet, Aurélien Chaline, Jean-François Franetich, Jean-Yves Brossas, Noémie Shahmirian, Olivier Silvie, Xavier Tannier, Renaud Piarroux

**Affiliations:** 1 Sorbonne Université, Inserm, Institut Pierre-Louis d’Epidémiologie et de Santé Publique, IPLESP, AP-HP, Groupe Hospitalier Pitié-Salpêtrière, Service de Parasitologie-Mycologie, 75013 Paris, France; 2 Sorbonne Université, AP-HP, Groupe Hospitalier Pitié-Salpêtrière, Service de Parasitologie-Mycologie, 75013 Paris, France; 3 Section Informatique et Communication, École Polytechnique Fédérale de Lausanne (EPFL), Lausanne, Suisse; 4 Sorbonne Université, Inserm, CNRS, Centre d’Immunologie et des Maladies Infectieuses, CIMI-Paris, 75013 Paris, France; 5 Sorbonne Université, Inserm, Université Sorbonne Paris Nord, Laboratoire d’Informatique Médicale et d’Ingénierie des Connaissances pour la e-Santé, LIMICS, 75006 Paris, France

**Keywords:** Computational biology and bioinformatics, Parasitology, Entomology

## Abstract

Vector control programmes are a strategic priority in the fight against malaria. However, vector control interventions require rigorous monitoring. Entomological tools for characterizing malaria transmission drivers are limited and are difficult to establish in the field. To predict *Anopheles* drivers of malaria transmission, such as mosquito age, blood feeding and *Plasmodium* infection, we evaluated artificial neural networks (ANNs) coupled to matrix-assisted laser desorption ionization-time of flight (MALDI-TOF) mass spectrometry (MS) and analysed the impact on the proteome of laboratory-reared *Anopheles stephensi* mosquitoes. ANNs were sensitive to *Anopheles* proteome changes and specifically recognized spectral patterns associated with mosquito age (0–10 days, 11–20 days and 21–28 days), blood feeding and *P. berghei* infection, with best prediction accuracies of 73%, 89% and 78%, respectively. This study illustrates that MALDI-TOF MS coupled to ANNs can be used to predict entomological drivers of malaria transmission, providing potential new tools for vector control. Future studies must assess the field validity of this new approach in wild-caught adult *Anopheles*. A similar approach could be envisaged for the identification of blood meal source and the detection of insecticide resistance in *Anopheles* and to other arthropods and pathogens.

## Introduction

Mosquito species that belong to the genus *Anopheles* have the capacity to transmit parasites such as *Plasmodium* species, which are the agents of malaria. These pathogens are transmitted to humans during the blood meal of an infected female *Anopheles* mosquito^1^. Despite global malaria control efforts, the disease persists, and approximately 405,000 deaths were estimated to have occurred globally in 2018 by the World Health Organization^2^. In addition, the increase in insecticide resistance among *Anopheles* mosquito populations worldwide is of considerable concern^3^. Vector control programmes are a strategic priority in the fight against malaria now more than ever^4^. However, malaria transmission and the efficacy of vector control interventions require rigorous monitoring.

Unfortunately, tools for characterizing the entomological drivers of malaria transmission, such as mosquito age or infection status, are limited and difficult to implement in the field^5^. Mosquito age can be estimated indirectly by the mark-release-recapture method for instance^6^. This approach consists of releasing marked mosquitoes (dye, radiolabels, dusts) and recapturing them at a series of time points. It is time-consuming, requires considerable human resources and the marking of a large number of mosquitoes to avoid bias. Another indirect method for the estimation of mosquito age is the morphological determination of ovariole dilatation to distinguish whether a female mosquito is nulliparous (has not yet laid eggs), and therefore likely to be young, or parous (has laid eggs), and therefore older^7^. It is based on the microscopic observation of modifications of the tracheoles that surround the ovaries. To establish *Plasmodium* infection rates in mosquitoes, the microscopy observation of salivary glands has been routinely performed in malaria-endemic countries^1^. However, microscopy methods are labourious and require fresh material and technical skills.

The improvement of the estimation of *Anopheles* drivers of malaria transmission in the field would have implications for vector control and thus malaria control. Alternative techniques have been developed for *Anopheles* age grading^5^. For instance, analyses of cuticular hydrocarbons^8^, protein profiling^9^, near-infrared and mid-infrared spectroscopy (NIRS and MIRS)^10,11^ and transcriptional profiling^12,13^ have been proposed. However, their utility for field-based monitoring programs remains largely untested. To detect *Plasmodium* in mosquitoes, enzyme-linked immunosorbent assays (ELISAs) targeting circumsporozoite protein and PCR techniques are routinely used^14^. However, the time required for the preparation of the samples and the cost involved can limit the use of these methods for extensive screening. Thus, there is a need for operationally attractive methods to assess *Anopheles* drivers of malaria transmission.

MALDI-TOF MS has been widely used for the species identification of bacteria^15^, fungi^16^, parasites^17^ and, more recently, arthropod vectors^18^. This proteomic tool is increasingly being employed not only in northern countries but also in disease-endemic countries. Indeed, this technique is robust, easy to use and the consumables that it requires are inexpensive. Protein profiling based on MALDI-TOF MS spectra has provided potential biomarkers of pathogen-infected arthropods^17^, antimicrobial resistance^15^ and closely-related *Anopheles* species^19^. Supervised machine-learning methods are a set of algorithms applied to already labelled data to learn a statistical model for pattern recognition, classification or prediction. This model must be able to generalize the learned task to new, unseen data. Artificial neural networks (ANNs) are a class of machine-learning algorithms. Deep ANNs such as convolutional neural networks are able to produce a reduced representation from sequences of elements (images as a sequence of pixels, text as a sequence of words). Several previous studies have demonstrated that ANNs can recognize informative patterns in mass spectra acquired from MALDI-TOF MS^20,21^, but this approach has never been tested for medical entomology applications.

To provide new tools to monitor entomological drivers of malaria transmission, we evaluated whether MALDI-TOF MS could provide a suitable input for ANNs to classify the spectral patterns of *Anopheles* biology. Using MALDI-TOF MS coupled with ANNs and laboratory-reared *Anopheles stephensi* that were either blood-fed or not and infected with *Plasmodium berghei* or uninfected*,* we evaluated the prediction of age, blood meal history and *Plasmodium* infection status*.* This paper presents the results of spectral classification performed by ANNs and the potential biomarkers obtained by protein profiling.

## Methods

### *Anopheles* rearing

Mosquitoes from a colony of *Anopheles stephensi* (line Nijmegen SDA500) were reared at Sorbonne University, Paris, France. In water trays, larval stages were reared at a temperature of 28 ± 1 °C and a relative humidity of 70%. Adults were transferred to incubators with a temperature of 20.8 ± 0.2 °C and a relative humidity of 70%. We analysed three categories of adult mosquitoes: (1) mosquitoes that did not receive a blood meal (unfed), (2) mosquitoes that received an uninfected blood meal (fed and uninfected) and (3) mosquitoes that received a *Plasmodium berghei* (GFP ANKA strain)-infected blood meal (infected)^22^. The transmission of rodent malaria in mosquitoes was conducted in strict accordance with Directive 2010/63/EU of the European Parliament and Council on the protection of animals used for scientific purposes. Protocols were approved by the local Ethical Committee Charles Darwin C2EA N°05, Sorbonne Université, Paris, France (approval #7475-2016110315516522). The characteristics of the mosquitoes in each dataset are presented in Tables 1 and 2.

**Table 1:** Mosquito characteristics in the whole dataset, experiment 1.

Mosquito age post-emergence (days)	0	3	6	10	11	14–15	17	20	21	27–28
Unfed (no. mosquitoes)	10	10	10	10	0	10	0	10	0	10
Fed and uninfected (no. mosquitoes)	0	0	0	0	10	10	10	0	10	10
Infected (no. mosquitoes)	0	0	0	0	10	10	10	0	10	10

**Table 2:** Mosquito characteristics in the whole dataset, experiment 2.

Mosquito age post-emergence (days)	0	3	6	10	11	14–15	17	20	21	27–28
Unfed (no. mosquitoes)	5	3	2	3	0	5	0	5	0	5
Fed and uninfected (no. mosquitoes)	0	0	0	0	5	5	5	0	5	5
Infected (no. mosquitoes)	0	0	0	0	5	5	5	0	5	5

### *Anopheles* feeding and sampling

All adult mosquitoes were sugar-fed ad libitum on a 10% sucrose solution. The mosquitoes were blood-fed at day 4 post-emergence on two anaesthetized mice after overnight starvation. We removed visibly unfed mosquitoes from the cage in the cohort of blood-fed females. For the cohort of infected mosquitoes, we infected mice 4 days prior to mosquito feeding via the i.p. injection of 10^7^ parasitized red blood cells infected with GFP-expressing *P. berghei* (PbGFP)^22^. We previously checked for gametocytemia and exflagellation in male gametocytes. Using fluorescence microscopy to observe PbGFP expression, we could visualize oocysts in the abdomen from approximately 3 days postfeeding and sporozoites in the salivary glands (thorax) from approximately 13 days postfeeding. Thus, we checked the mosquito samples for infection at each age point. We recorded the relative intensity of fluorescence for each mosquito, and we discarded nonfluorescent *Anopheles* (abdomen and/or thorax).

### Sample preparation for MALDI-TOF MS

Mosquitoes of various chronological ages (expressed in calendar days) and physiological stages (blood feeding, infection, oviposition) were processed during two independent experiments (Tables 1, 2). For each mosquito category, the mosquitoes were killed at different age points post-emergence by freezing at − 20 °C for 30 min. We dissected the mosquitoes into 4 body parts (head, thorax with wings, abdomen and legs) immediately after killing (experiment 1, n = 170) or after storage for up to 5–10 months at − 20 °C (experiment 2, n = 78). The head was severed from the thorax by a complete cut, leaving the salivary glands in the thorax. Directly after dissection, we performed protein extraction from the mosquitoes’ body parts according to a previously published protocol [23]. The protein extracts were then deposited onto a steel plate and covered with an alphacyano-4-hydroxycinnamic acid matrix. To ensure reproducibility of the results, we acquired spectra from four replicates of each protein extract, as previously reported^23,24^. We considered each spectrum as a single input.

### Acquisition of mass spectra

Mass spectrum acquisition was performed with a Microflex LT (Bruker France SAS) using the default acquisition parameters. The spectra were acquired in linear mode in ion-positive mode at a laser frequency of 60 Hz and mass range of 2–20 kDa. The data were automatically acquired using AutoXecute in FlexControl v3.4 software (Bruker France SAS) with the default parameters and exported into Maldi Biotyper v4.1, ClinProTools v.3.0 software and Flex Analysis v3.4 software for data processing and spectrum analysis.

## Classification of mass spectra

### Data preparation

To build the model, we used a training dataset to fit the parameters. To predict the responses from the fitted model and to evaluate the performance, we used a test dataset that contained new and unseen data. We trained three separate ANNs with different classification targets: age-grading, past blood meal and *P. berghei* infection. For each classification target, we performed training and testing using all the mosquito categories (unfed, fed and uninfected, infected). Each body part was tested in a separate dataset. To avoid blood interactions, we included fed mosquitoes from day 7 post-blood meal. We first used a dataset of 680 spectra to build and test each network model (experiment 1, Table 3). For each mosquito category, spectra were acquired with the same instrument during independent analysis using two MALDI-TOF plates. For each acquisition, we split the data into a training dataset (50%) and a test dataset (50%) according to the plate that was used (one or two). For unfed mosquitoes, the second plate was acquired in a different period. Then, we verified that the ANN classification was not biased by the date of acquisition. For this purpose, we processed frozen mosquitoes from previous sampling points into the same target to simultaneously acquire spectra from each different category of *Anopheles* (experiment 2, Table 4). The dataset of 312 spectra was split temporally into training (60%) and test datasets (40%).

**Table 3:** Spectral characteristics in the training and test datasets for each mosquito body part, experiment 1.

	Age grading		Past blood meal		*Plasmodium* infection	
	Training	Test	Training	Test	Training	Test
Unfed (no. spectra)	140	140	140	140	140	140
Fed and uninfected (no. spectra)	100	100	100	100	100	100
Fed and infected (no. spectra)	100	100^a^	100	100^a^	100	100^a^

^a^One leg spectrum was missing in the leg dataset.

**Table 4:** Spectral characteristics in the training and test datasets for each mosquito body part, experiment 2.

	Age grading		Past blood meal		*Plasmodium* infection	
	Training	Test	Training	Test	Training	Test
Unfed (no. spectra)	80	32	80	32	80	32
Fed and uninfected (no. spectra)	60^a^	40	60^a^	40	60^a^	40
Fed and infected (no. spectra)	60	40	60	40	60	40

^a^One leg spectrum was missing in the leg dataset.

### Machine learning

**Figure 1:**
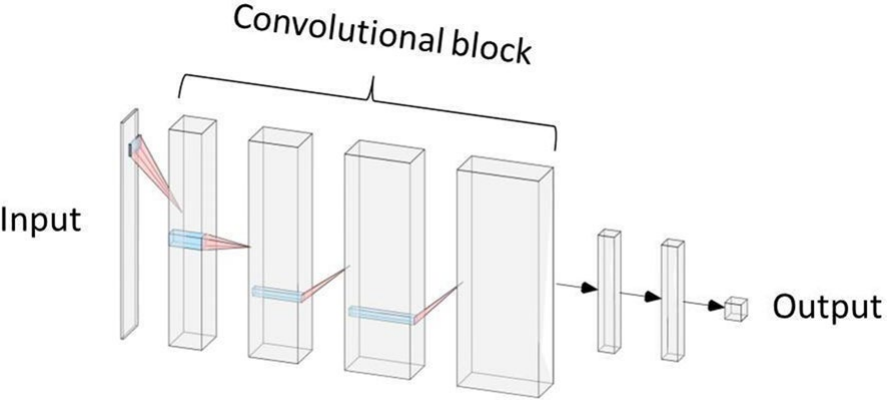
Architecture of the artificial neural network used for the prediction of *Anopheles* categories.

We preprocessed the spectra by smoothing using the moving average method and removing the baseline. As input for the ANNs, an entry of 10,000 distinct values in a single dimension was reduced to 100 values by searching for local maxima. These 100 values corresponded to the 100 highest peaks to avoid background noise. The classifier was a convolutional neural network composed of 4 convolutional blocks (Fig. 1). The convolutional block consisted of a convolutional layer, followed by a batch normalization layer, then a ReLU activation layer, a pooling layer and a dropout layer, with a dropout rate of 0.3. The numbers of successive convolutional filters by layer were 8, 16, 32, 64, with a stride of 1, with filter sizes of 50, 25, 10 and 10. Finally, there were 3 dense layers with sizes of 140, 130 and 2. The used loss is a standard categorical cross-entropy loss, but weighted to obtain a better balance between false positives and false negatives (penalty factor of 5). We then conducted optimization with the Adam optimizer^25^ and a learning rate of 0.001. The networks were trained for 100 continuous iterations, at which point the training was stopped. To reduce the variance due to the randomness of the optimization algorithm, we trained 9 neural networks in parallel, and they all voted to provide the final result.

### Classification performance evaluation

We performed a quantitative evaluation of the classification performance of the three trained ANNs based on the output of the test dataset. The output variables were the number of spectra in each labelled class. Sensitivity (SS) and specificity (SP) were computed as the rates of correctly classified spectra in the positive and negative labelled classes, respectively. The positive predictive value (PPV), negative predictive value (NPV) and classification accuracy (Acc) are a combination of sensitivity and specificity. The mean accuracy was calculated from the results of 10 different training runs of the model.

$$ \begin{aligned} & SS = \frac{TP}{{TP + FN}}; \quad SP = \frac{TN}{{TN + FP}} \\ & PPV = \frac{TP}{{TP + FP}}; \quad NPV = \frac{TN}{{TN + FN}} \\ & Acc* = \left( {TP + TN} \right)/\left( {TP + TN + FP + FN} \right) \\ \end{aligned} $$

*$\left( {Acc = \frac{\sum TP}{{Total}} } \right)$ if more than 2 labelled classes (age prediction).

### Protein profiling

To compare the profiles between categories of *Anopheles*, we loaded the mass spectra into ClinProTools 3.0 software. We generated a peak list in the 2–20 kDa mass range. The parameter settings for peak picking in average spectra were as follows: resolution of 8,000 ppm; noise threshold of 1.00 (arbitrary intensity unit); maximum peak shift of 1,000 ppm; and match to calibration peaks of 30%. According to the mass range, the mass resolving power was estimated at 5–10 Da. Then, we analysed the spectra with the Peak Statistic tool using the t-test/ANOVA sort mode to generate a list of peaks with differences in intensity. We analysed the 25 most discriminant peaks (*p* value < 0.05).

## Results

Two experiments were performed in order to validate our results. Because it was the first study that assessed ANN classification of *Anopheles* mass spectra, we prioritized the using of fresh mosquitoes from insectary colony. Therefore, experiment 1 tested ANN classification of spectra from freshly killed mosquitoes, when each category and each age was acquired independently and immediately after killing. As each category of mosquito was acquired independently, a bias of classification could have occurred. Thus, to validate experiment 1 results, we performed a simultaneous acquisition of each category and age, with mosquitoes stored at − 20 °C (experiment 2). To more closely approximate field conditions, the training and testing of the model were carried out with the 3 categories of mosquitoes (unfed, fed and uninfected, infected) at different ages and times post blood-feeding.

## Classification of mass spectral profiles using ANNs

As ANNs successfully discriminated spectra categories whether categories were acquired independently or simultaneously, we have shown that ANNs classification was truly supported by physiological changes and was not biased by experimental conditions. The classification was not impacted by the age of the mosquitoes (data not shown) or by the mode of spectrum acquisition. ANNs could recognize informative patterns in mass spectra acquired from MALDI-TOF MS during two different experiments. Spectra of different categories obtained during the same acquisition (experiment 2) were successfully classified by the ANNs, showing that the classification was based on physiological status. Spectra of each category and each age obtained independently (experiment 1) were also successfully classified, which indicated robustness, especially as the test set for unfed *Anopheles* was obtained during an independent manipulation conducted several months from the other experiments. We present the complete results for the anatomic parts that provided the best ANN classification performance for the test set. For age predictions, the ANN classification performance using the thorax is presented in Table 5. For blood meal prediction, the ANN classification performance using the legs or thorax is presented in Table 6. For *Plasmodium* infection prediction using the legs or thorax, the ANN classification performance is presented in Table 7. The results for other anatomic parts are provided in the supplementary data (Supplementary Tables S1–S5).

**Table 5:** Classification performance of the artificial neural network trained for age prediction using the thorax.

Thorax	Experiment 1 (n = 340)			Experiment 2 (n = 112)		
Age category (days)	0–10	11–20	21–28	0–10	11–20	21–28
TP	57	117	69	6	52	25
FP	40	52	5	2	17	10
TN	220	128	235	102	31	62
FN	23	43	31	2	12	15
SS (%)	71	73	69	75	81	63
SP (%)	85	71	98	98	65	86
PPV (%)	59	69	93	75	75	71
NPV (%)	91	75	88	98	72	81
Acc (%)	81	72	89	96	74	78

*TP* true positive, *FP* false positive, *TN* true negative, *FN* false negative, *SS* sensitivity, *SP *specificity, *PPV* positive predictive value, *NPV* negative predictive value, *Acc* accuracy.

**Table 6:** Classification performance of the artificial neural network trained for past blood meals.

	Experiment 1		Experiment 2	
	Legs (n = 339)	Thorax (n = 340)	Legs (n = 112)	Thorax (n = 112)
TP	92	70	23	24
FP	39	82	7	6
TN	160	118	73	74
FN	48	70	9	8
SS (%)	66	50	72	75
SP (%)	80	59	91	93
PPV (%)	70	46	77	80
NPV (%)	77	63	89	90
Acc (%)	74	55	86	88

*TP* true positive, *FP* false positive, *TN* true negative, *FN* false negative, *SS* sensitivity, *SP* specificity, *PPV* positive predictive value, *NPV* negative predictive value, *Acc* accuracy.

**Table 7:** Classification performance of the artificial neural network trained for the detection of *Plasmodium* infection.

	Experiment 1		Experiment 2	
	Legs (n = 339)	Thorax (n = 340)	Legs (n = 112)	Thorax (n = 112)
TP	87	57	20	33
FP	54	109	16	16
TN	186	131	56	56
FN	12	43	20	7
SS (%)	88	57	50	83
SP (%)	78	55	78	78
PPV (%)	62	34	56	67
NPV (%)	94	75	74	89
Acc (%)	81	55	68	79

*TP* true positive, *FP* false positive, *TN* true negative, *FN* false negative, *SS* sensitivity, *SP* specificity, *PPV* positive predictive value, *NPV* negative predictive value, *Acc* accuracy.

### Aging

For age prediction, the best classification performance was observed using the thorax (Table 5). There was no imbalance in performance between the age groups. The results were robust, with comparable classification accuracies between experiment 1 and experiment 2. The mean accuracy ± SD values for the 3 age groups during experiment 1 and experiment 2 were 72.1 ± 2.5% and 73.4 ± 4%, respectively. The thorax was the most interesting anatomic part, as it showed the best accuracy and NPV, particularly for the extreme age categories of 0–10 days (accuracy = 96%, NPV = 98%, experiment 2) and 21–28 days (accuracy = 89%, NPV = 88%, experiment 1). The age category of 11–20 days showed a lower accuracy and NPV (accuracy = 72%, NPV = 75%, experiment 1). The legs, head and abdomen exhibited a classification performance close to that of the thorax, but the accuracy was lower for the 11–20-day and 21–28-day categories (see Supplementary Tables S1–S3 online). The abdomen presented the lowest accuracy, down to 60% (experiment 1) and 67% (experiment 2) for the 11–20-day and 21–28-day categories.

### Blood feeding and *Plasmodium* infection

For the blood meal and *Plasmodium* infection predictions, the best classification performance was observed using the legs and the thorax (Tables 6, 7). The best mean accuracy ± SD values for the blood meal and *P. berghei* infection predictions were 88.8 ± 2.7% (thorax, experiment 2) and 78.1 ± 1.9% (legs, experiment 1), respectively. The lowest classification performance was observed using the head. Both the abdomen and head provided insufficient sensitivity, resulting in low accuracy for both the blood meal and infection predictions (see Supplementary Tables S4 and S5).

From experiment 1 to experiment 2, the mean accuracy of blood meal anteriority prediction using the legs rose from 78.3 ± 2.9% to 87.3 ± 2.7%, respectively. However, the mean accuracy of *P. berghei* infection prediction using the legs decreased from 78.1 ± 1.9% to 65.0 ± 2.6%. In contrast, when using the thorax, the performance for past blood meal and infection predictions increased. The mean accuracy for blood meal anteriority prediction using the thorax increased from 58.6 ± 2.4% to 88.8 ± 2.7%, and the mean accuracy for *P. berghei* infection prediction rose from 57.6 ± 3.6 to 75.9 ± 3.9%.

## Protein profiling

**Figure 2:**
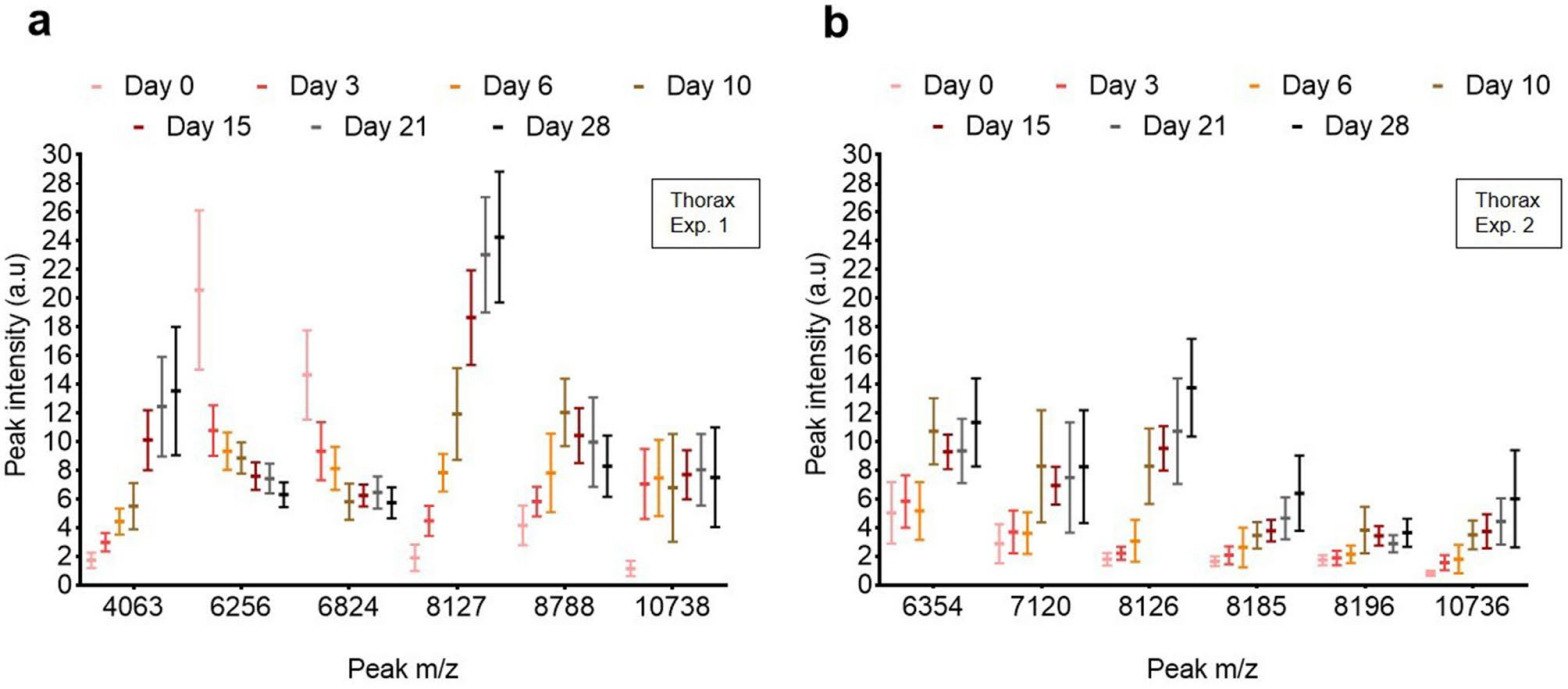
Box and whisker plot showing 6 peaks of distinct m/z (Da) with varying intensity for the complete dataset (line: mean, whiskers: standard deviation). (**a**, **b**) Spectra obtained at 7 age points using the thorax of *Anopheles stephensi* (non-blood-fed) during (**a**) experiment 1 and (**b**) experiment 2.

**Figure 3:**
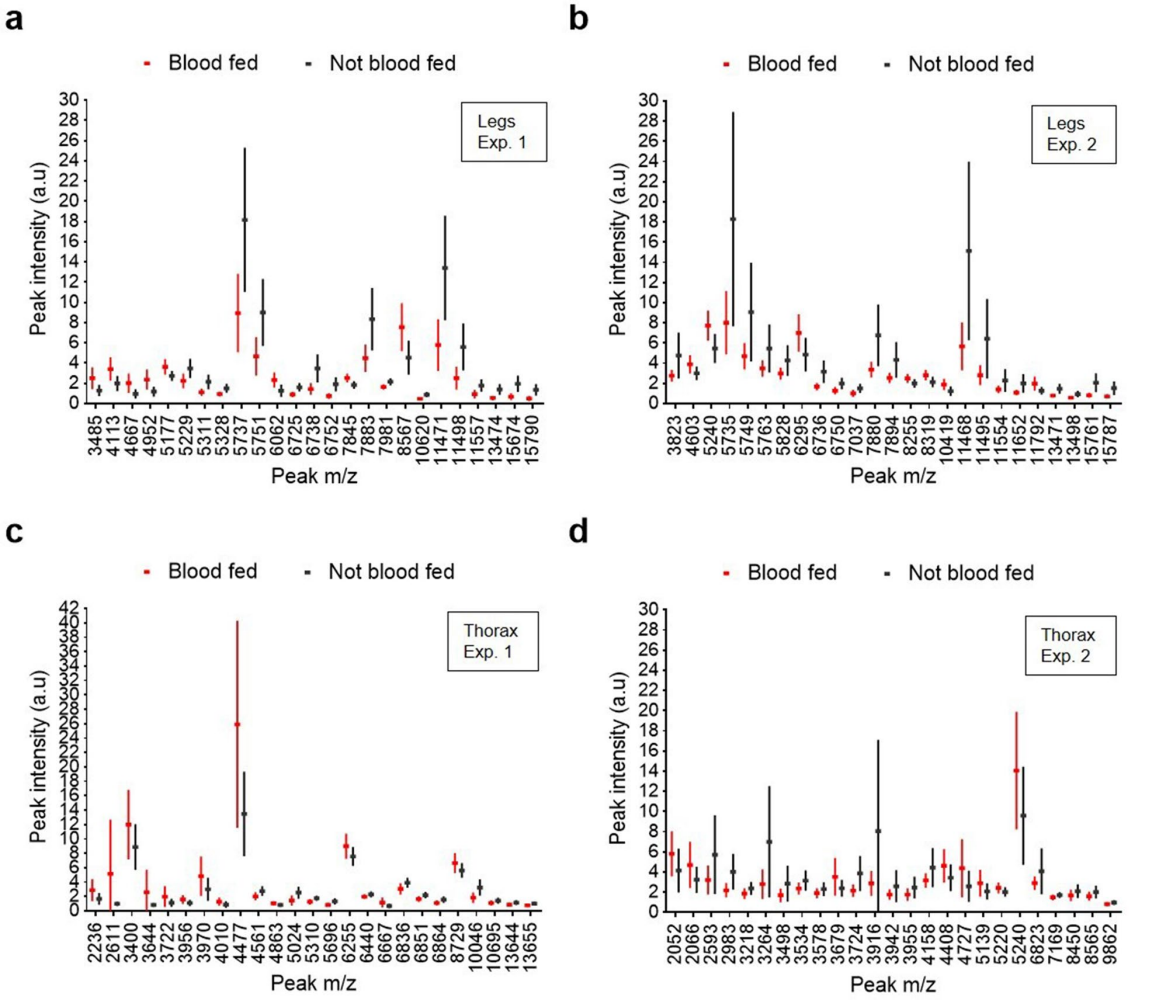
Box and whisker plots demonstrating varying intensities of 25 peaks of distinct m/z (Da) for the complete dataset (line: mean, whiskers: standard deviation). Each peak corresponds to blood-fed (red) or non-blood-fed (black) mosquitoes of the same ages. (**a**, **b**) Spectra obtained using the legs of *Anopheles stephensi* that were blood fed (not infected) or were not blood fed during (**a**) experiment 1 and (**b**) experiment 2. (**c**, **d**) Spectra obtained using the thorax of *Anopheles stephensi* that were blood fed (not infected) or were not blood fed during (**c**) experiment 1 and (**d**) experiment 2.

**Figure 4:**
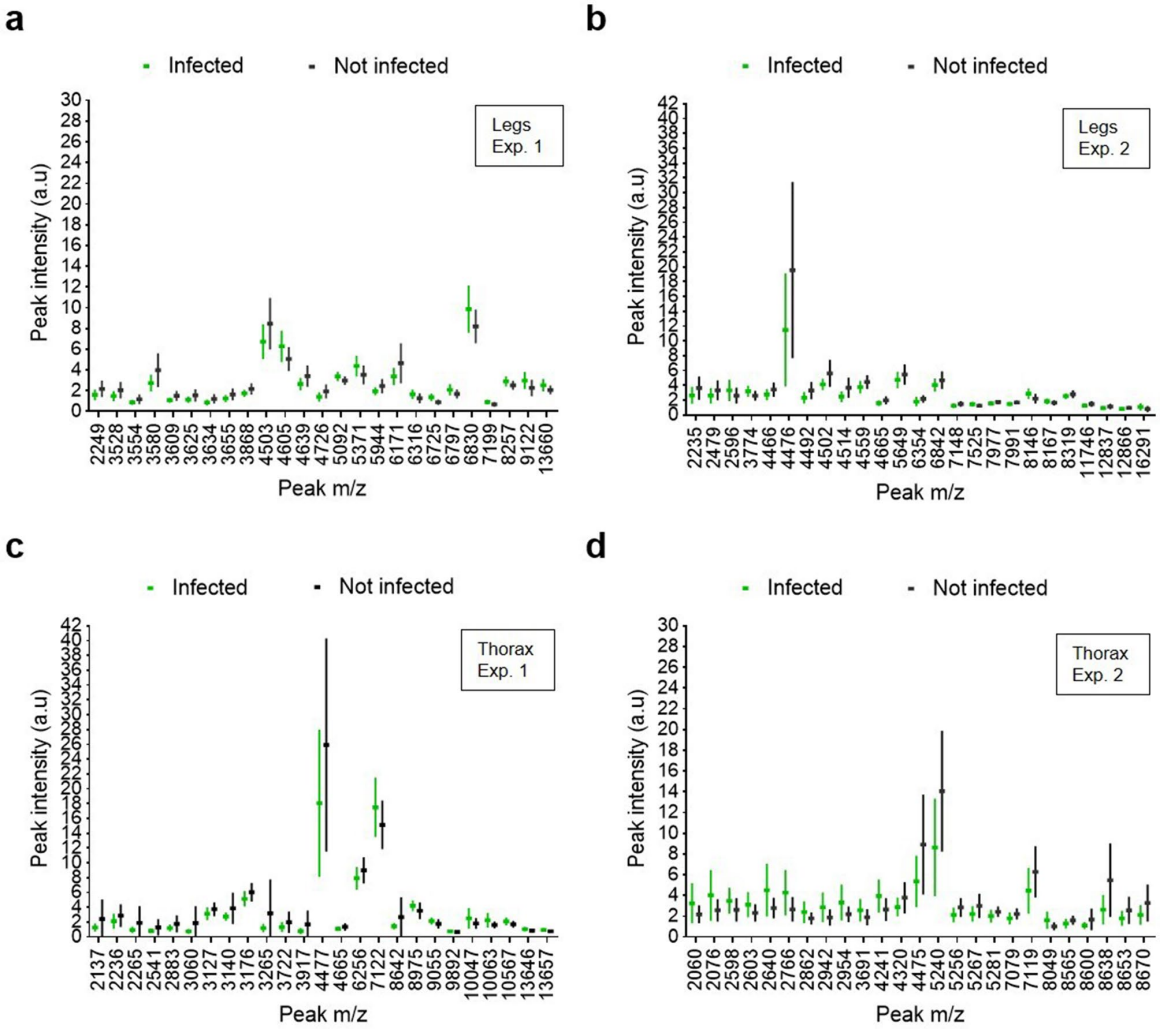
Box and whisker plots demonstrating varying intensities of 25 peaks of distinct m/z (Da) for the complete dataset (line: mean, whiskers: standard deviation). Each peak corresponds to infected (green) or uninfected (black) blood-fed mosquitoes of the same ages. (**a**, **b**) Spectra obtained using the legs of *Anopheles stephensi* that were infected or not infected by *Plasmodium berghei* during (**a**) experiment 1 and (**b**) experiment 2. (**c**, **d**) Spectra obtained using the thorax of *Anopheles stephensi* that were infected or not infected by *Plasmodium berghei* during (**c**) experiment 1 and (**d**) experiment 2.

The mass spectra were very similar, without any apparent, consistently reproducible single peak(s) correlated with each category. However, we observed variations in peak intensity that may provide interesting biomarkers (Figs. 2, 3, 4). Representative mass spectra protein profiles are provided in the Supplementary data (Supplementary Fig. S1–S5). No significant changes of peak intensity and profile were observed between mosquitoes stored at − 20 °C from 5 to 10 months.

### Aging

During aging, we observed peaks with a decreasing or increasing intensity in the thorax spectrum profiles of *An. stephensi* (Fig. 2, Table 8). We observed a constant peak linked to aging in experiment 1 (Fig. 2a) and experiment 2 (Fig. 2b) with a similar mass (m/z 8,127 and 8,126, respectively). Another peak present in both experiments showed a better correlation with aging after freezing in experiment 2 (m/z 10,736).

**Table 8:** List of the characteristic peaks and intensity variation from day 0 to day 28, using the thorax of *Anopheles stephensi.*

Mass m/z (Da)	Intensity
4,063	↗
6,256	↘
6,354	↗
6,824	↘
7,120	↗
8,127^a^	↗
8,185	↗
8,196	↗
8,788	↗
10,738^a^	↗

^a^Peak present in both experiments 1 and 2.

### Blood feeding

Following a blood meal, proteomic comparative analysis of blood-fed and non-blood-fed mosquitoes revealed peaks with varying intensities in the leg and thorax spectrum profiles (Fig. 3, Table 9). Using the legs, at least 4 discriminant peaks (m/z 5,737, m/z 5,751, m/z 11,471, m/z 11,498) corresponding to 2 proteins (double charged) were observed in experiment 1 (Fig. 3a) and in experiment 2 (m/z 5,735, m/z 5,749, m/z 11,468, m/z 11,495) (Fig. 3b). Using the thorax, we observed 3 discriminant peaks (m/z 2,611, m/z 3,644, m/z 4,477) in experiment 1 (Fig. 3c). In experiment 2 (Fig. 3d), we observed other discriminant peaks (m/z 2,593, m/z 2,983, m/z 3,264, m/z 3,916). Following frozen storage (experiment 2), we observed more peaks of interest for both the legs and thorax (Fig. 3b, d, respectively).

**Table 9:** List of the characteristic peaks and intensity variation following blood-feeding, using the legs and the thorax of *Anopheles stephensi.*

Legs		Thorax	
Mass m/z (Da)	Intensity	Mass m/z (Da)	Intensity
3,823	↘	2,593	↘
5,240	↗	2,611	↗
5,737^a^	↘	2,983	↘
5,751^a^	↘	3,264	↘
6,738^a^	↘	3,644	↗
7,883^a^	↘	3,724	↘
7,894^a^	↘	3,916	↘
8,567	↗	4,477	↗
11,471^a^	↘	6,823	↘
11,498^a^	↘	10,046	↘

^a^Peak present in both experiments 1 and 2.

### *Plasmodium* infection

Following *Plasmodium berghei* infection of *An. stephensi,* proteomic comparative analysis of infected and uninfected blood-fed mosquitoes revealed peaks with varying intensities in the leg and thorax spectrum profiles (Fig. 4, Table 10). The variations in peak intensity were small, and most discriminant peaks exhibited a low intensity. Using the legs, at least 4 discriminant peaks (m/z 3,580, m/z 4,503, m/z 6,171, m/z 6,380) were observed in experiment 1 (Fig. 4a). In experiment 2, one discriminant peak (m/z 4,476) observed using the legs (Fig. 4b) was also present using the thorax (Fig. 4d). Using the thorax, we observed at least 4 other discriminant peaks in experiment 2 (m/z 2,640, m/z 5,240, m/z 7,119, m/z 8,638). Following frozen storage (experiment 2), we observed more peaks of interest for the thorax (Fig. 4d) but not for the legs (Fig. 4b).

**Table 10:** List of the characteristic peaks and intensity variation following infection by *Plasmodium berghei*, using the legs and the thorax of *Anopheles stephensi.*

Legs		Thorax	
Mass m/z (Da)	Intensity	Mass m/z (Da)	Intensity
2,235	↘	2,060	↗
3,580	↘	2,076	↗
4,476	↘	2,640	↗
4,503^a^	↘	2,766	↗
4,514	↘	3,060	↘
4,605	↗	3,265	↘
4,639	↘	4,241	↗
5,371	↗	4,476^a^	↘
6,171	↘	5,240	↘
6,830	↗	8,638^a^	↘

^a^Peak present in both experiments 1 and 2.

## Discussion

Using laboratory-reared *An. stephensi,* spectra from three cohorts of mosquito and four body parts (head, thorax with wings, legs, abdomen) were analysed with different overlapping and complex *Anopheles* biology targets: age, past blood meal and *P. berghei* infection. These *Anopheles* biology patterns are malaria transmission drivers useful for vector control. We have shown for the first time that MALDI-TOF MS spectra represent a suitable input for ANNs to classify *Anopheles* spectra. This proteomic study of *Anopheles* also revealed the presence of biomarkers showing intensity variations.

Proteomic analysis of *Anopheles* vectors are already performed to identify new targets for parasite or vector control and new diagnostic biomarkers^26^. However, this approach has been underutilized in comparison to genomic or transcriptomic methods, potentially due to limited access to high-end mass spectrometers and complex workflows. Advances in computational biology have permitted the detection and recognition of complex spectral patterns using the simplest workflows, such as MALDI-TOF MS. In microbiology, the machine-learning analysis of MALDI-TOF MS spectra has enabled the differentiation of strains that are resistant and sensitive to antimicrobials such as azole in *Candida albicans*^27^, methicillin in *Staphylococcus aureus*^28^ or carbapenem in *Klebsiella pneumoniae*^29^. A recent study used a similar approach to distinguish between *E. coli* and *Shigella* species^21^. In clinical pathology, this strategy was applied to the blood serum proteome to predict the presence of monoclonal gammopathy of undetermined significance^30^ and to discriminate between multiple myeloma patients and healthy donors^20^. In entomology, MALDI-TOF MS coupled with machine learning approaches has been tested to distinguish closely related *Anopheles* vector species^19^. However, to our knowledge, the use of ANNs coupled with MALDI-TOF MS for the investigation of *Anopheles* vector biology has not been assessed, which would expand the field of proteomics applications.

The average age of *Anopheles* female mosquito population is an important determinant of the likelihood of malaria transmission. Indeed, only the oldest mosquitoes in a population are responsible for *Plasmodium* transmission, as the parasite requires 9–14 days of incubation inside female mosquito vectors before it becomes infectious to humans, once its sporozoites are present in the salivary glands^5^. We have shown that age-dependent protein expression patterns can be specifically recognized by ANNs, allowing age-related spectral classification. The best age prediction results were obtained using the thorax. For 0–10-day-old mosquitoes, we obtained a good accuracy (up to 96%) and NPV (up to 98%), enabling a good estimation of the proportion of mosquitoes older than 10 days, which are more likely to be infectious. However, the overall mean accuracy was lower (approximately 73%) and was maybe due to the lower performance of the intermediate category (11–20 days). Indeed, it presented the largest sample size and was probably characterized by intermediate physiological changes. Using a combination of two-dimensional difference gel electrophoresis (2D-DIGE), MALDI-TOF/TOF, and LC–MS, aging-related proteome changes were observed in the thorax and head across the three age groups of *An. stephensii* (9, 17 and 34 days old) and four age groups of *An. gambiae* (1, 9 and 17 days old)^9^. The authors showed that approximately 4% of *Anopheles* proteins displayed robust age-dependent regulation, including metabolic proteins, stress-related molecular chaperones, and cuticular proteins. If field-validated, this age-grading approach will be useful for assessing the efficacy of vector control measures, showing a reduction in age populations^5,10,11^. Moreover, a reduction in mosquito survival has been shown to be the most effective measure for reducing malaria transmission^5^.

Recent blood meals are usually assessed by the visual examination of the abdominal state or the stage of blood digestion^1^. However, within 48–72 h in tropical and subtropical climates, mosquitoes have digested the blood and oviposited [1], making it impossible to visually determine a past blood meal. We have shown that the proteomic changes that occur in response to a blood meal (≥ 7 days delay) can be specifically recognized by ANNs, allowing the successful classification of spectra from 7 to 25 days post-blood meal. The best results of blood meal anteriority prediction were obtained using the legs and thorax after storage of the mosquitoes by freezing at − 20 °C. The overall mean accuracy was acceptable and comparable between the two body parts, at 87% and 89% for the legs and the thorax, respectively. In addition, the NPVs were high, at 89% and 90% for the legs and thorax, respectively. Thus, this method allows a good estimation of the proportion of unfed mosquitoes. This approach should also be evaluated to look for host-specific proteomic changes following blood meal. Indeed, PCR success rate for host identification, drops sharply after blood meal digestion^31,32^, from 84.5 to 25% for the most digested ones^32^. This decreased in success rate has been shown to occur about 30–36 h after feeding^32^. The proteomic responses of *Anopheles* to blood feeding have been previously explored by comparative analysis of the midgut proteins of sugar- and blood-fed *An. albimanus* using 2D-PAGE^33^. The author identified several molecules with altered abundance after blood intake, including enzymes related to innate immunity, the cytoskeleton, stress responses, signalling, digestion, detoxification and metabolism. The anteriority of the blood meal is an indicator of how often mosquitoes feed and provides information on the ability of a vector to transmit malaria. This method could lead to a new estimation of the proportion of blood-fed mosquitoes, which is assumed to decrease under effective vector control measures.

The evidence of *Anopheles* infection by *Plasmodium* is necessary to confirm the role of a given species as a vector, and the proportion of mosquitoes with sporozoites in the salivary glands (thorax) is a determinant of the capacity of malaria transmission^1^. We have shown that proteomic changes in response to *Plasmodium* infection can be specifically recognized by ANNs, allowing spectral classification according to infectious status. The best results of *P. berghei* infection prediction were obtained using fresh leg and thorax specimens after storage at − 20 °C. The overall mean accuracy was acceptable and comparable between the two body parts, at 78% and 76% for the legs and thorax, respectively. In addition, the NPVs were high, at 94% and 89% for the legs and thorax, respectively. Thus, this method allows good estimation of the proportion of uninfected mosquitoes. This is interesting as one of the drawbacks of PCR approaches for the detection of mosquito *Plasmodium* infection is the presence of non-specific amplifications. Indeed, a previous study has reported a specificity of 60% with a real-time qPCR targeting the COI gene^34^. Similarly, the ELISA that detects surface circumsporozoite protein has been shown to overestimate the infection rate due to false positive antigen reactions^35^. A previous study distinguished MALDI-TOF MS spectra of *An. stephensi* according to *P. berghei* infection status using the cephalothorax, without machine learning^24^. Nevertheless, spectra were acquired only at day 18 post-infection, and the dataset was small (only 100 spectra in total). Proteomic studies investigating the mosquito immune response to *Plasmodium* infection in the salivary glands^36,37^, haemolymph^38,39^, head^40^ and brain^41^ showed differential expression of proteins related to metabolism, synaptic transmission, signalling, and cytoskeletal remodelling. Changes in the haemolymph proteome could explain the detection of *Plasmodium* infection using the legs, which are not assumed to host the parasite. Our approach provides a new estimation of the proportion of *Plasmodium*-infected mosquitoes (legs and thorax), which would decrease under effective vector control measures. A proteomic characterization of the biomarkers from the thorax would help to determine the presence of biomarkers specific of the salivary glands’ infection, in order to estimate the entomological inoculation rate.

Proteomic profiling did not show specific distinct peaks between the categories to be classified but showed variations in peak intensity, thereby revealing potential biomarkers related to mosquito age, past blood meal and *Plasmodium* infection status. These results support an ANN classification based on physiological variations. Similarly, in a previous study involving MALDI-TOF MS coupled with the use of ANNs^20^, the authors did not observe distinct peaks specific to a category and postulated that ANNs were sensitive to small variations in the peak intensities. Additionally, we have shown that freezing mosquitoes at − 20 °C can modify the spectra. Some proteins might have been degraded at − 20 °C, generating new biomarkers and eliminating others. However, the stability of protein profiles from frozen mosquitoes indicates that the ANNs classification of frozen mosquitoes was not biased by modifications induced by storage over the time (5–10 months at − 20 °C). Overall, there was an increase in discriminant biomarkers after freezing, and ANN classification was improved, except for infection prediction using the legs. For example, using the thorax, blood meal and *Plasmodium* infection could be predicted effectively only after freezing. Similarly, the MALDI-TOF characterization of *Cryptosporidium* showed that a freeze–thaw procedure increased spectral biomarkers and improved sensitivity^42^. The authors observed a loss of some spectral biomarkers and a gain of others, suggesting a biomolecule degradation or separation by the freeze–thaw procedure. Another MALDI-TOF MS study showed that freezing preprocessing at − 20 °C yielded better identification results for mycobacteria, possibly by improving protein extraction^43^. In addition, the simultaneous acquisition of frozen mosquitoes may also have decreased the mass spectra variability and increased the detection of small variations of intensities. To ensure feasibility of the method for future *Anopheles* spectra library constructions, freezing should be recommended as well as simultaneous acquisition of each category. It would facilitate the implementation of this new approach in the field.

This is a proof of concept, and despite showing good specificity, the sensitivity could be further optimized. The biomarkers described here should be used for the further optimization of ANN classification and to obtain a deeper understanding of *Anopheles* biology, using high-end mass spectrometers such as LC–MS/MS systems. As the aim of the study was to build a field application, we used a simple workflow not adapted to protein characterization and we were not in capacity to compare our biomarkers to those previously published. Nevertheless, the specificity of the biomarkers needs to be further evaluated as important changes were observed between the two experiments. The preprocessing of the spectra could also be improved. Indeed, we selected the 100 highest peaks to avoid background noise, but we identified the presence of low-intensity biomarkers, especially for the *Plasmodium* infection target. In addition, despite the presence of discriminant biomarkers, the sensitivity of the ANNs was sometimes insufficient. This may derived from the presence of overlapping peaks between the mosquito categories and from an insufficient reproducibility of the observed biomarkers, as peak picking was performed on the average spectrum. Surprisingly, the abdomen did not yield an acceptable classification performance for infection and blood meal predictions. Insufficient spectral reproducibility and interference between overlapping physiological parameters, such as the blood meal and infection could explain this disparity. The optimization of spectral acquisition parameters could decrease the spectral variability and increase the intensity of peaks of interest. Only the most reproducible and informative peaks identified by protein profiling could then be selected as an input of ANNs, as previously performed by Deulofeu et al.^20^, who selected 28 informative peaks. Other network architectures can also be tested, such as recurrent networks, which retain the entire sequence in their memory and can then better take into account the succession of the peaks and lower signals. Similarity metric learning would allow a comparison between two spectra, one of which is known. Even if separated body parts have shown performance disparities, the accuracy of ANN classification of MALDI-TOF mass spectra using the entire mosquito is worth to be tested to facilitate field analysis. Finally, field validation needs to be performed and the applicability to wild-caught *Anopheles* and *Plasmodium* species need to be assessed, especially if *Plasmodium* infection is to be detectable at the parasite densities observed in the field. As kinetics of aging may vary between field and laboratory, semi-field conditions will have to be reproduced. To train the ANNs with mosquitoes of pre-determined age, larvae will have to be collected on the field and bred to the adult stage, using the F1 generation^11^.

## Conclusions

We evaluated the use of ANNs coupled with MALDI-TOF MS to predict *Anopheles* drivers of malaria transmission. We have shown that ANNs are sensitive to proteome changes and specifically recognize spectral patterns linked to *Anopheles* biology, such as aging, blood feeding and *Plasmodium* infection. We obtained good prediction accuracies and negative predictive values for the test dataset, but sensitivity should be further optimized. Peaks with intensity variations offer discriminant biomarkers that might be recognized by ANNs. This proof of concept extends the field of the proteomics applications of MALDI-TOF MS, providing new tools for vector control. In a context of malaria elimination, a large proportion of old mosquitoes with blood meal anteriority could reflect insufficient vector control measures and a risk of transmission resurgence following new malaria cases. A similar approach could be applied to the identification of blood meal source and the detection of insecticide resistance in *Anopheles* and to other arthropods and pathogens. Future studies must assess the field validity of this new approach to wild-caught adult *Anopheles* replicated across field sites.

## Supplementary information

Supplementary Information 1.

Supplementary Information 2.

Supplementary Information 3.

Supplementary Information 4.

Supplementary Information 5.

Supplementary Information 6.

## Data Availability

The datasets generated and/or analysed during the current study are available from the corresponding author upon reasonable request.
