# Supplementary figures and images for: Prediction of malaria transmission drivers in Anopheles mosquitoes using artificial intelligence coupled to MALDI-TOF mass spectrometry
Source: Sci Rep. 2020 Jul 9;10:11379. doi: 10.1038/s41598-020-68272-z (PMC7347643; doi:10.1038/s41598-020-68272-z)

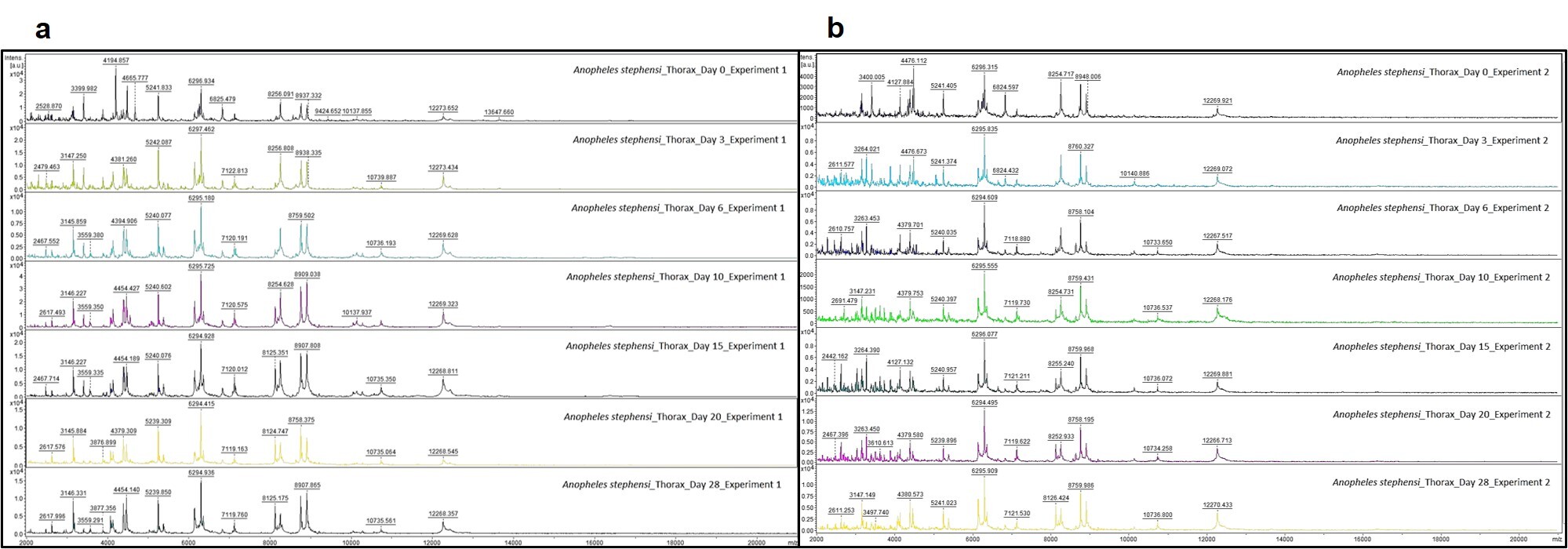

Supplement: Supplementary file 1 — Supplementary Information 1. [file 41598_2020_68272_MOESM1_ESM.jpg]

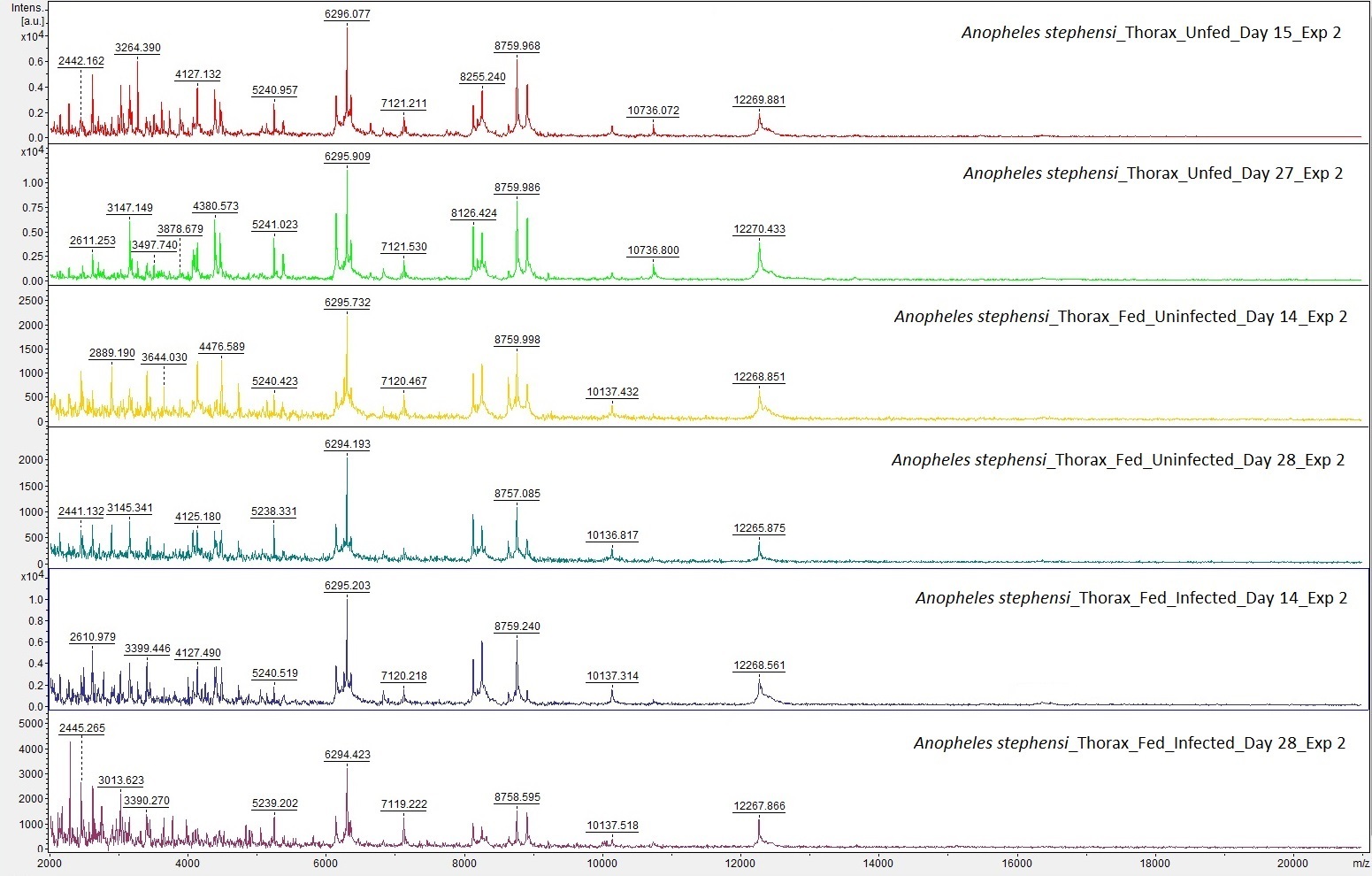

Supplement: Supplementary file 2 — Supplementary Information 2. [file 41598_2020_68272_MOESM2_ESM.jpg]

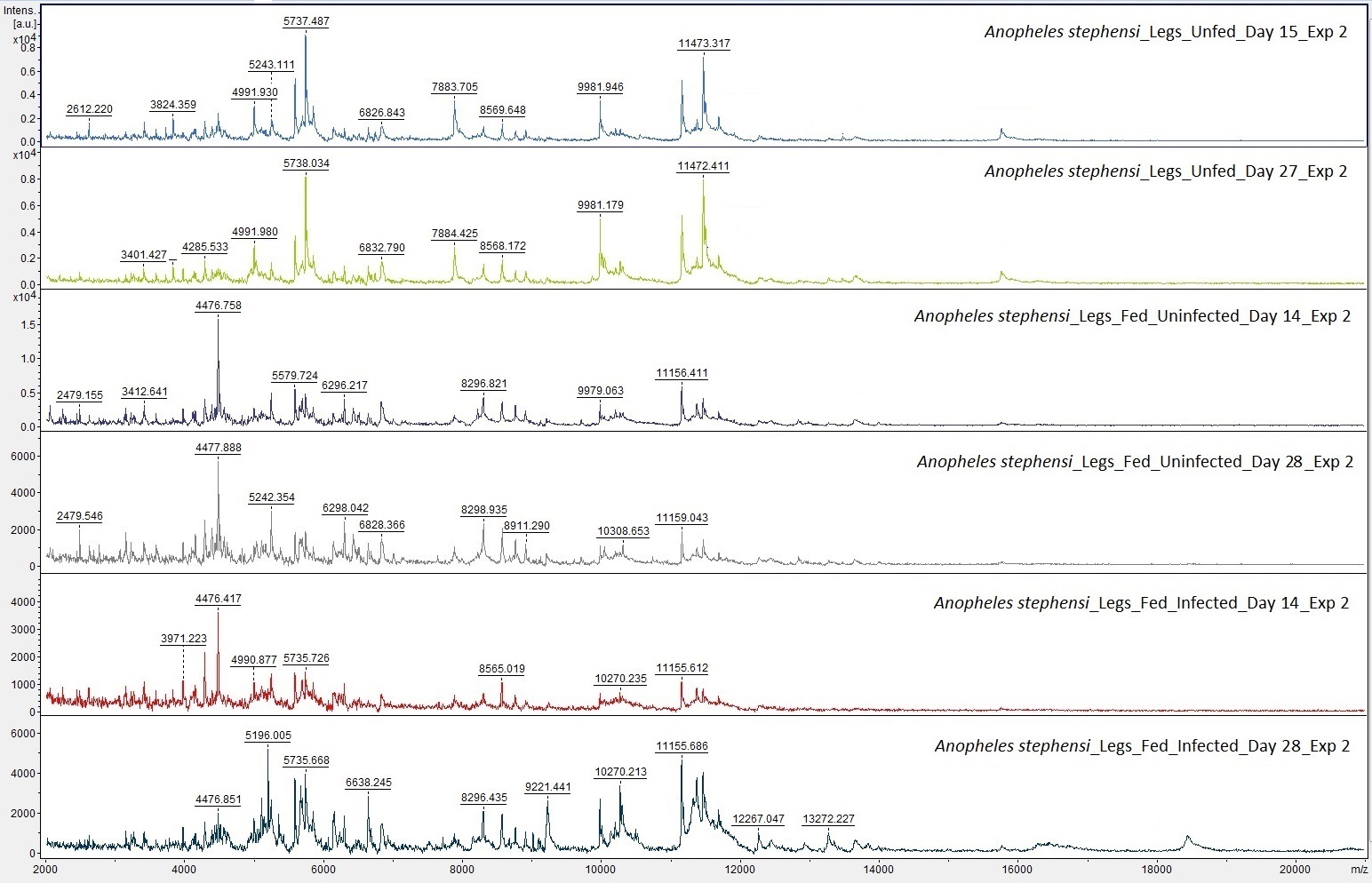

Supplement: Supplementary file 3 — Supplementary Information 3. [file 41598_2020_68272_MOESM3_ESM.jpg]

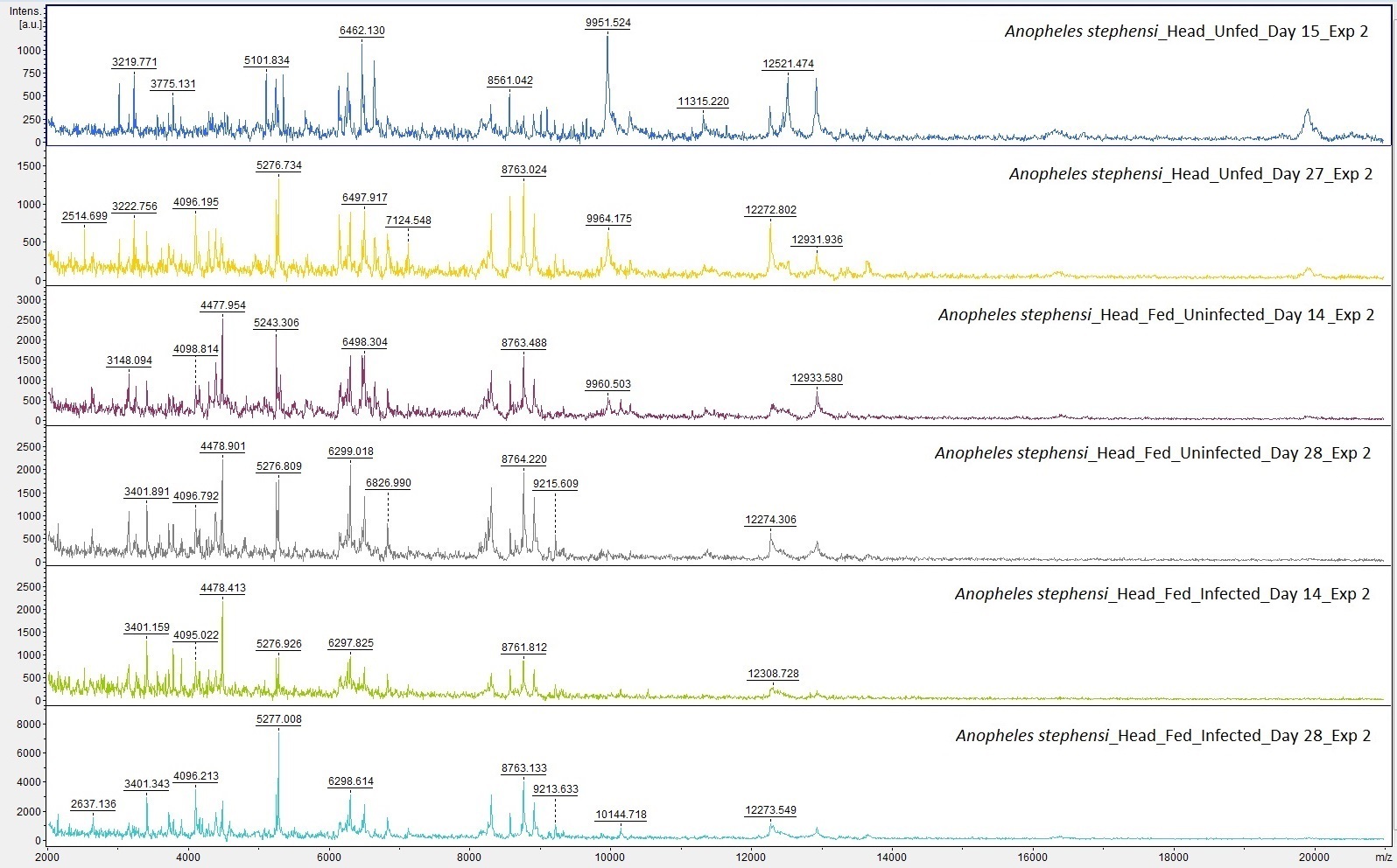

Supplement: Supplementary file 4 — Supplementary Information 4. [file 41598_2020_68272_MOESM4_ESM.jpg]

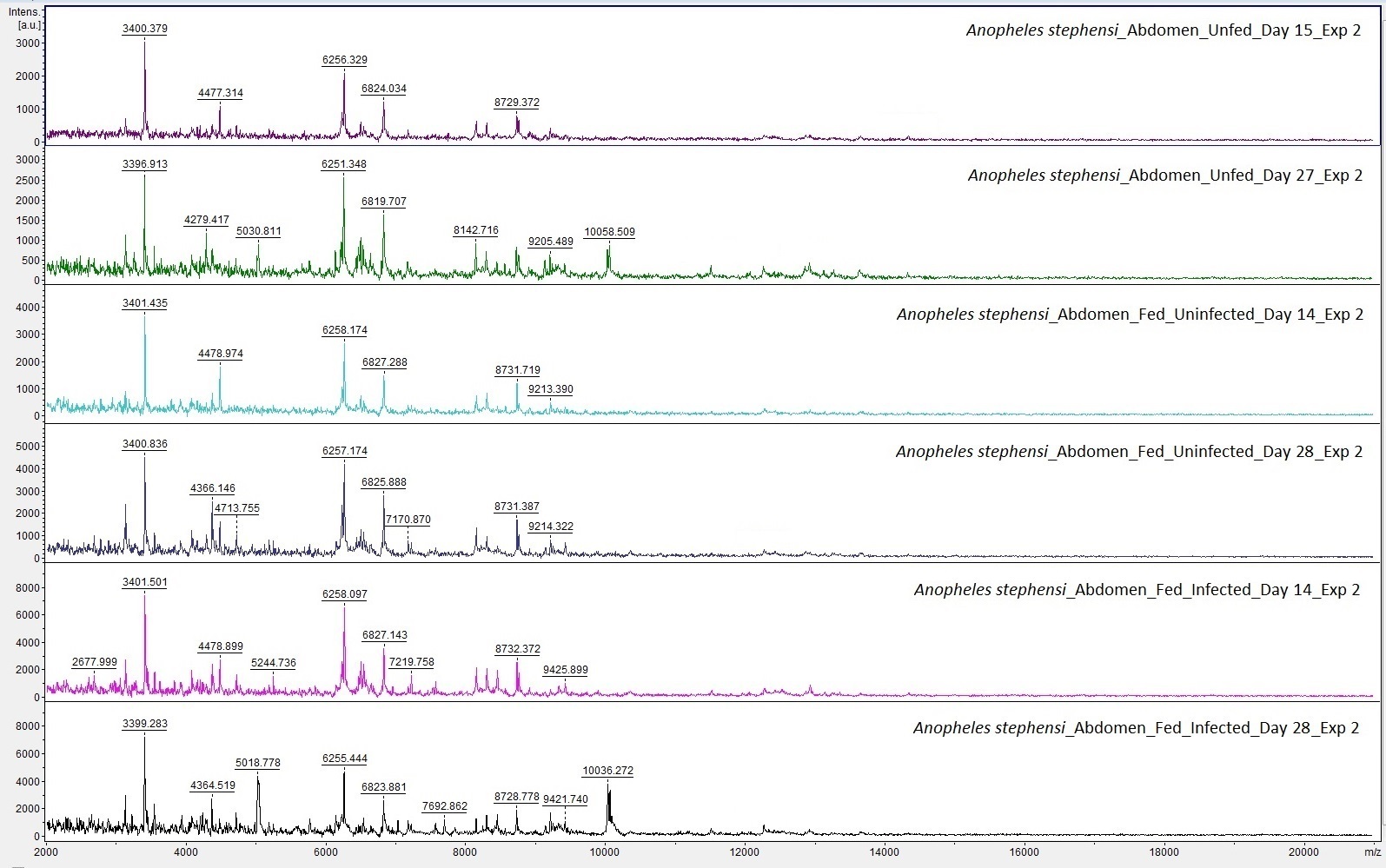

Supplement: Supplementary file 5 — Supplementary Information 5. [file 41598_2020_68272_MOESM5_ESM.jpg]
