# Supplementary material for: Prediction of malaria transmission drivers in Anopheles mosquitoes using artificial intelligence coupled to MALDI-TOF mass spectrometry
Source: Sci Rep. 2020 Jul 9;10:11379. doi: 10.1038/s41598-020-68272-z (PMC7347643; doi:10.1038/s41598-020-68272-z)
Supplement: Supplementary file 6 — Supplementary Information 6. [file 41598_2020_68272_MOESM6_ESM.pdf]

Prediction of malaria transmission drivers in *Anopheles* mosquitoes using artificial intelligence coupled to MALDI-TOF mass spectrometry

Cécile Nabet[1*], Aurélien Chaline[2,3], Jean-François Franetich[4], Jean-Yves Brossas[2], Noémie Shahmirian[2], Olivier Silvie[4], Xavier Tannier[5], Renaud Piarroux[1]

Table S1. Classification performance of the artificial neural network trained for age prediction using legs.

Legs	Experiment 1 (n=339)			Experiment 2 (n=112)		
Age category (days)	0-10	11-20	21-28	0-10	11-20	21-28
TP	42	146	32	8	51	19
FP	16	91	12	2	20	12
TN	243	89	227	102	28	60
FN	38	13	68	0	13	21
SS (%)	53	92	32	100	80	48
SP (%)	94	49	95	98	58	83
PPV (%)	72	62	73	80	72	61
NPV (%)	86	87	77	100	68	74
Acc (%)	84	69	76	98	71	71

TP: true positive, FP: false positive, TN: true negative, FN: false negative, SS: sensitivity, SP: specificity, PPV: positive predictive value, NPV: negative predictive value, Acc: accuracy.

Table S2. Classification performance of the artificial neural network trained for age prediction using the head.

Head	Experiment 1 (n=340)			Experiment 2 (n=112)		
Age category (days)	0-10	11-20	21-28	0-10	11-20	21-28
TP	45	104	74	8	53	18
FP	24	60	33	2	22	9
TN	236	120	207	102	26	63
FN	35	56	26	0	11	22
SS (%)	56	65	74	100	83	45
SP (%)	91	67	86	98	54	88
PPV (%)	65	63	69	80	71	67
NPV (%)	87	68	89	100	70	74
Acc (%)	83	66	83	98	71	72

TP: true positive, FP: false positive, TN: true negative, FN: false negative, SS: sensitivity, SP: specificity, PPV: positive predictive value, NPV: negative predictive value, Acc: accuracy.

Table S3. Classification performance of the artificial neural network trained for age prediction using the abdomen.

Abdomen	Experiment 1 (n=340)			Experiment 2 (n=112)		
Age category (days)	0-10	11-20	21-28	0-10	11-20	21-28
TP	35	128	38	7	52	14
FP	18	103	18	4	24	11
TN	242	77	222	100	24	61
FN	45	32	62	1	12	26
SS (%)	44	80	38	88	81	35
SP (%)	93	43	93	96	50	85
PPV (%)	66	55	68	64	68	56
NPV (%)	84	71	78	99	67	70
Acc (%)	81	60	76	96	68	67

TP: true positive, FP: false positive, TN: true negative, FN: false negative, SS: sensitivity, SP: specificity, PPV: positive predictive value, NPV: negative predictive value, Acc: accuracy.

Table S4. Classification performance of the artificial neural network trained for past blood meals using the abdomen and head.

	Experiment 1		Experiment 2	
	Abdomen (n=340)	Head (n=340)	Abdomen (n=112)	Head (n=112)
TP	69	53	16	12
FP	16	25	7	20
TN	184	175	73	55
FN	71	87	16	25
SS (%)	49	38	50	32
SP (%)	92	88	91	73
PPV (%)	81	68	70	38
NPV (%)	72	67	82	69
Acc (%)	74	67	79	60

TP: true positive, FP: false positive, TN: true negative, FN: false negative, SS: sensitivity, SP: specificity, PPV: positive predictive value, NPV: negative predictive value, Acc: accuracy.

Table S5. Classification performance of the artificial neural network trained for the detection of *Plasmodium* infection using the abdomen and head.

	Experiment 1		Experiment 2	
	Abdomen (n=340)	Head (n=340)	Abdomen (n=112)	Head (n=112)
TP	61	71	24	8
FP	80	95	21	32
TN	160	145	51	54
FN	39	29	16	18
SS (%)	61	71	60	31
SP (%)	67	60	71	63
PPV (%)	43	43	53	20
NPV (%)	80	83	76	75
Acc (%)	65	64	67	55

TP: true positive, FP: false positive, TN: true negative, FN: false negative, SS: sensitivity, SP: specificity, PPV: positive predictive value, NPV: negative predictive value, Acc: accuracy.

Figure S1. Representative mass spectra protein profiles using the thorax of *Anopheles stephensi* (unfed), at 7 age points during **a** experiment 1 and **b** experiment 2.

Figure S2. Thorax mass spectra protein profiles of the 3 categories of *Anopheles stephensi* (unfed, fed and uninfected, infected) at 2 ages and times post blood-feeding, experiment 2.

Figure S3. Legs mass spectra protein profiles of the 3 categories of *Anopheles stephensi* (unfed, fed and uninfected, infected) at 2 ages and times post blood-feeding, experiment 2.

Figure S4. Head mass spectra protein profiles of the 3 categories of *Anopheles stephensi* (unfed, fed and uninfected, infected) at 2 ages and times post blood-feeding, experiment 2.

Figure S5. Abdomen mass spectra protein profiles of the 3 categories of *Anopheles stephensi* (unfed, fed and uninfected, infected) at 2 ages and times post blood-feeding, experiment 2.
